# Transcranial magnetic stimulation and motor overflow: a systematic review in neurological disorders

**DOI:** 10.3389/fnins.2026.1819449

**Published:** 2026-06-18

**Authors:** Gabrielly Santos Pereira, João Eduardo De Araújo, Gustavo Henrique De Mello Rosa, Kelly Zhang, Marcelo Lourenço Da Silva, Luciano Maia Alves Ferreira

**Affiliations:** 1 Neuropsychobiology and Motor Control Laboratory, Department of Health Sciences, Ribeirão Preto Medical School, University of São Paulo, Ribeirão Preto, São Paulo, Brazil; 2 Laboratory of Neuroscience, Neuromodulation and Study of Pain (LANNED), Federal University of Alfenas (UNIFAL-MG), Alfenas, Brazil; 3 Neuromodulation and Pain Unit (NeuroPain), Egas Moniz Interdisciplinary Research Center (CiiEM), Almada, Portugal

**Keywords:** cortical excitability, dystonia, interhemispheric inhibition, mirror movements, motor overflow, neurorehabilitation, stroke, transcranial magnetic stimulation

## Abstract

Motor overflow, a neuromotor phenomenon characterized by involuntary activation of muscles during voluntary movement, reflects impairments in interhemispheric and intracortical inhibition and is commonly observed in conditions such as stroke, cerebral palsy, dystonia, and Parkinson’s disease. Transcranial Magnetic Stimulation (TMS) is a non-invasive neuromodulatory technique with potential to modulate the cortical excitability underlying overflow-related dysfunctions. This systematic review aimed to evaluate the efficacy of TMS in reducing motor overflow across neurological populations. Seven randomized controlled trials (RCTs) were included, encompassing participants with stroke, cerebral palsy, Parkinson’s disease, and focal hand dystonia. Protocols varied in frequency (1–10 Hz), target area (M1, SMA, PSC, PMC), and design (e.g., crossover, combined protocols with constraint-induced therapy or cerebellar stimulation; Motor overflow was directly assessed in only two studies, one involving Parkinson’s disease and one involving dystonia, thereby limiting cross-study comparisons. Therefore, evidence supporting a direct effect of TMS on motor overflow remains preliminary and condition-specific. In dystonia, high-frequency rTMS (10 Hz) targeting the primary somatosensory cortex led to significant reductions in overflow, demonstrated by handwriting kinematics. In Parkinson’s disease, although physiological modulation (reduced IHI) was observed after SMA stimulation, mirror movements remained unchanged. In stroke and cerebral palsy populations, overflow was not directly evaluated, though improvements in motor function, spasticity, and cortical excitability were consistently reported. Risk of bias was low in most included studies, although gaps in allocation reporting and standardization of outcome measures were noted. This review highlights the potential of TMS, particularly high-frequency protocols, to modulate motor overflow in focal dystonia. However, the lack of targeted assessment in other neurological conditions suggests a critical need for future trials with standardized protocols and specific outcome measures focused on overflow to clarify the therapeutic role of TMS in rehabilitation.

## 1 Introduction

Motor overflow refers to a neuromotor phenomenon characterized by involuntary and simultaneous muscle activation in regions of the body that are not directly involved in the execution of a voluntary motor task. This process reflects a failure in interhemispheric and intracortical inhibitory mechanisms, resulting in the unwanted propagation of motor activity to adjacent or contralateral areas (Mayston et al., 1999). These unintended activations often mirror or accompany the intended movement and are particularly prevalent in individuals with neurological impairments such as stroke, cerebral palsy, and Parkinson’s disease (Cincotta and Ziemann, 2008; Koerte et al., 2010). Although occasionally present in healthy individuals, especially children during motor development, the persistence or reemergence of overflow in pathological conditions suggests a disruption in inhibitory motor control mechanisms (Mayston et al., 1999).

The underlying pathophysiology of motor overflow is multifactorial, involving reduced interhemispheric inhibition, impaired integrity of the corticospinal tract, and compensatory recruitment of alternative motor pathways such as the reticulospinal tract (Xu et al., 2017; Chen et al., 2018). When the primary motor cortex and its descending projections fail to finely regulate neural excitability, an imbalance between excitation and inhibition emerges within the motor system. Reduced interhemispheric inhibition facilitates the spread of motor commands to unintended regions, while corticospinal damage compromises selective motor output. In this context, the central nervous system increasingly relies on secondary motor pathways that support gross motor execution but lack precision, leading to involuntary activations and excessive muscle synergies (Deconinck et al., 2015; Xu et al., 2017).

Motor overflow has important clinical implications, as it negatively affects motor performance and functional independence across neurological conditions. In stroke survivors, mirror movements and associated reactions have been associated with impaired hand function and reduced performance in tasks requiring fine motor control (Kimberley et al., 2010; Ejaz et al., 2018). In children with cerebral palsy, persistent mirror movements compromise bimanual coordination and interfere with daily activities (Koerte et al., 2010). In Parkinson’s disease, overflow may manifest as unintended limb or facial movements during fine motor tasks, contributing to functional disability (Quattrone et al., 2023). Collectively, these findings indicate that motor overflow is not merely an epiphenomenon of motor impairment, but rather a clinical marker of dysregulated neural control with direct consequences for quality of life and rehabilitation outcomes (Cincotta and Ziemann, 2008).

Traditional rehabilitation approaches, including physiotherapy and task-specific motor training, can improve overall motor performance but often fail to directly suppress involuntary motor activity (Araneda et al., 2022; Rosa et al., 2025). This limitation underscores a critical therapeutic gap: current rehabilitative strategies rarely target the cortical and subcortical mechanisms that sustain pathological motor overflow. Addressing this gap requires neuromodulatory approaches capable of directly influencing neural excitability and inhibitory control circuits.

In this context, transcranial magnetic stimulation (TMS) has emerged as a powerful non-invasive tool for probing and modulating motor system function. TMS delivers magnetic pulses to the cortex, allowing investigation of interhemispheric connectivity, intracortical inhibition, and motor control mechanisms (Lefaucheur et al., 2020). Beyond its diagnostic utility, TMS has demonstrated therapeutic potential by selectively increasing or decreasing cortical excitability depending on stimulation parameters, making it particularly attractive for correcting maladaptive motor network reorganization (Murase et al., 2004).

Importantly, the field of neuromodulation has shifted in recent years toward mechanism-driven, circuit-specific interventions rather than symptom-based approaches alone. Randomized trials using transcranial direct current stimulation (tDCS) in developmental dyslexia and repetitive TMS (rTMS) in affective disorders have demonstrated that targeting specific dysfunctional networks can yield disorder-specific improvements, reinforcing the importance of identifying precise neurophysiological targets (Kazemi et al., 2025; Rezaei et al., 2025). Despite this paradigm shift, motor overflow has not been systematically examined as a primary neuromodulation outcome, even in populations in which it is clinically prominent (Hao et al., 2013; Chen et al., 2018).

Importantly, TMS allows selective targeting of cortical regions implicated in motor overflow, including the primary motor cortex (M1), supplementary motor area (SMA), and prefrontal regions. Stimulation of the SMA, for instance, has been shown to reduce mirror movements in children with unilateral cerebral palsy (Sun et al., 2023). Similarly, inhibitory stimulation of the contralesional M1 has been associated with improved motor selectivity and reduced involuntary activation in stroke populations (Chen et al., 2019; Dai et al., 2022). These findings suggest that motor overflow may be modifiable through circuit-specific neuromodulation, but the evidence remains fragmented and inconsistently reported.

While multiple systematic reviews have evaluated TMS for general motor recovery—particularly after stroke—they have not isolated motor overflow as a distinct behavioral and neurophysiological construct. This distinction is critical, as improvements in global motor scores do not necessarily imply restoration of selective motor control or normalization of interhemispheric inhibition. The absence of a focused synthesis specifically addressing motor overflow represents a significant gap in the literature and limits the development of targeted rehabilitation strategies.

At the same time, it is important to recognize that much of the existing TMS literature in stroke and cerebral palsy has been designed to evaluate general motor recovery, even in populations in which motor overflow is clinically evident. This creates an interpretative challenge, as functional gains cannot be assumed to reflect overflow modulation in the absence of direct measurement. Consequently, the present review examines both studies that directly quantified motor overflow and studies that investigated motor and neurophysiological outcomes in populations where motor overflow is a clinically relevant phenomenon.

Therefore, a systematic review dedicated to TMS and motor overflow is both timely and necessary. The present review aims to evaluate the effectiveness of TMS in modulating motor overflow in neurological conditions by synthesizing evidence from randomized controlled trials and controlled experimental studies. By critically distinguishing between studies that directly measure motor overflow and those that assess related motor and neurophysiological outcomes, this review seeks to clarify the current state of evidence, identify methodological gaps, and inform future neuromodulation-based rehabilitation strategies targeting motor control dysregulation.

## 2 Methods

### 2.1 Study design

This systematic review evaluates the effects of Transcranial Magnetic Stimulation (TMS) on motor overflow in individuals with neurological conditions. The review followed the Preferred Reporting Items for Systematic Reviews and Meta-Analyses (PRISMA) 2020 guidelines (Page et al., 2021) and adhered to all 27 PRISMA checklist items, including structured reporting of eligibility criteria, search strategy, study selection, risk of bias assessment, and synthesis methods. The protocol was prospectively registered in PROSPERO (CRD420251117629), and no deviations from the registered protocol occurred during the review process (Table 1).

**Table 1:** Characteristics of included studies.

Study	Study design	Population/condition	TMS protocol	Primary outcome measures	Outcome measure	Effect direction	Duration of effect	Motor overflow change
Du et al. (2016)	RCT	Hemiplegic after first-ever stroke (*n* = 69)	rTMS: 3 Hz (affected) or 1 Hz (unaffected), 1,200 pulses, 80–120% rMT	Motor recovery (FMA, BI, mRS); cortical excitability (MEP, RMT)	Fugl-Meyer Assessment, Medical Research Council scale, Barthel Index, modified Rankin Scale, resting motor threshold, motor evoked potential amplitude/latency	Improved motor scores, increased excitability	Up to 2 weeks	No direct overflow measure; targets interhemispheric imbalance potentially linked to overflow
Li et al. (2021)	RCT	Stroke with hemiplegia (*n* = 90)	1 Hz rTMS (M1) + cTBS (cerebellum); 80% rMT/AMT, 1000–1,200 pulses	Motor function (FMA, MBI); spasticity (MAS)	Fugl-Meyer Assessment, Modified Barthel Index, Modified Ashworth Scale	Improved function, reduced spasticity	4 weeks	Overflow not measured; dual stimulation may influence cortical rebalancing mechanisms related to overflow
Sung et al. (2013)	RCT	Chronic hemiplegic stroke (*n* = 54)	1 Hz rTMS (contralesional M1), iTBS (ipsilesional M1), or both; sham controls	Motor recovery (FMA, WMFT, grip strength); cortical excitability (MEP, RMT)	Motor outcomes, cortical excitability	Improved motor outcomes	20 sessions	Overflow not directly assessed; bilateral modulation may affect interhemispheric imbalance contributing to overflow
Klingels et al. (2018)	RCT	Hemiparetic cerebral palsy (*n* = 45, age 6–19)	1 Hz rTMS (contralesional M1), 20 min/day; constraint therapy or both	Upper limb function (AHA, movement time, grip strength); neurophysiology (SICI, ICF)	Assisting Hand Assessment, movement time, short-interval intracortical inhibition, intracortical facilitation, motor evoked potential, grip strength	Improved Assisting Hand Assessment, neurophysiology predicted response	6 months	Overflow not measured, but inhibitory cortical markers (SICI) may reflect underlying overflow-related mechanisms
Sharples et al. (2014)	RCT	Parkinson’s disease with/without mirror movements (*n* = 21)	5 Hz rTMS, 750 pulses, 110% AMT, supplementary motor area (SMA)	Mirror activation (sEMG); interhemispheric inhibition (IHI)	Unified Parkinson’s Disease Rating Scale III, interhemispheric inhibition, excitability index	Interhemispheric inhibition reduced, no Unified Parkinson’s Disease Rating Scale change	Immediate	Direct overflow evaluation via sEMG; IHI reduced but mirror movements unchanged
Rajak et al. (2017)	RCT	Spastic cerebral palsy (*n* = 45, age 2–16)	5 Hz or 10 Hz rTMS, 1,500 pulses, M1, 15 min/day for 20 sessions	Hand function (QUEST); improvements observed, especially in older children	Quality of Upper Extremity Skills Test	Improved hand function	Post 20 sessions	Overflow not measured directly; functional gains may indirectly suggest improved voluntary motor control
Bukhari-Parlakturk et al. (2025)	RCT	Writer’s cramp dystonia (*n* = 12)	10 Hz rTMS, 4,000 pulses, 90% rMT, primary somatosensory cortex (PSC) / premotor cortex (PMC), cross-over	Handwriting kinematics (peak acceleration); motor overflow reduction observed post PSC stimulation	Dystonia scales, kinematic writing	Improved writing, reduced dysfluency	One session	Overflow directly assessed via handwriting metrics; significant session-level reduction in peak acceleration

### 2.2 Eligibility criteria

The eligibility criteria were defined using the PICO framework:

Population: Children, adolescents, or adults diagnosed with neurological disorders known to present motor overflow, including stroke (ischemic or hemorrhagic), cerebral palsy, Parkinson’s disease, dystonia, and spinal cord injury. Studies were eligible if they either directly assessed motor overflow (e.g., mirror movements, associated reactions, EMG-defined overflow) or evaluated motor and neurophysiological outcomes closely related to overflow regulation, such as interhemispheric inhibition, cortical excitability, motor selectivity, or sensorimotor integration.

Intervention: Studies employing TMS (including repetitive TMS [rTMS], intermittent or continuous theta burst stimulation [iTBS or cTBS], or single/paired-pulse paradigms) with therapeutic intent to modulate motor overflow. Interventions had to report stimulation parameters, including site, frequency, intensity, and the number of sessions.

Comparison: Sham TMS, no intervention, conventional care, or alternative TMS parameters.

Outcomes: The primary outcome was a reduction in motor overflow, assessed using surface electromyography (sEMG), standardized clinical scales (e.g., the Mirror Movement Scale), or structured observation protocols. Secondary outcomes included motor function (e.g., Fugl-Meyer Assessment [FMA], Box and Block Test [BBT]), changes in cortical excitability (e.g., MEPs, RMT), and adverse events.

Study design: Only randomized controlled trials (RCTs) published in peer-reviewed journals were eligible. Case reports, reviews, editorials, and conference abstracts were excluded from the analysis.

### 2.3 Information sources

Searches were conducted in the following electronic databases: PubMed, Embase, Scopus, LILACS, and MEDLINE via BVS. The search covered publications from August 1, 2010 to August 1, 2025, and included articles in English, Spanish, or Portuguese. Additional studies were identified through manual search of reference lists and consultation with field experts.

No automation tools were used for study selection. Grey literature was not included due to the requirement for peer-reviewed methodological quality.

### 2.4 Search strategy

The search strategy was constructed using a combination of MeSH terms and free-text keywords related to TMS and motor overflow. Boolean operators AND and OR were applied as follows:

(“Transcranial Magnetic Stimulation” OR “rTMS” OR “Theta Burst Stimulation” OR “Non-invasive Brain Stimulation”).

AND (“Motor Overflow” OR “Mirror Movements” OR “Involuntary Movement” OR “Associated Reaction”).

AND (“Stroke” OR “Cerebral Palsy” OR “Parkinson Disease” OR “Spinal Cord Injury” OR “Neurological Disorders”).

Search strings were adapted to the specific indexing systems of each database. The full reproducible search strategies, including field tags and filters, are provided in the PROSPERO registration.

### 2.5 Study selection

All identified studies were imported into Rayyan for duplicate removal and screening. Two independent reviewers (GSP and MLS) screened titles and abstracts, followed by full-text analysis based on the inclusion criteria. Disagreements were resolved through discussion or by a third reviewer (LMAF). Inter-reviewer agreement was assessed qualitatively, and discrepancies were documented. Reviewer agreement was ensured through independent screening and consensus-based resolution of discrepancies, although a formal quantitative measure of agreement (e.g., Cohen’s kappa) was not calculated. The selection process was documented using a PRISMA flow diagram.

### 2.6 Data extraction

Data were extracted independently by two reviewers (GSP and MLS) using a standardized form, including:

Study characteristics: author, year, country, design, sample size.

Population: diagnosis, age, sex.

Intervention: TMS parameters (site, frequency, intensity, sessions).

Comparator: sham, none, or alternative TMS.

Outcomes: type and timing of overflow measures, functional assessments, cortical excitability, adverse events.

Data extraction forms were pilot-tested prior to full extraction to ensure consistency and completeness. When required data were missing, authors were contacted directly.

### 2.7 Risk of bias assessment

The RoB 2.0 tool (Cochrane Collaboration) was used to assess risk of bias in RCTs across five domains: randomization process, deviations from intended interventions, missing outcome data, measurement of outcomes, and selection of the reported result. Each domain was rated as “low risk,” “some concerns,” or “high risk” (Sterne et al., 2019). Two reviewers (GSP and MLS) independently performed the risk-of-bias assessment. Any disagreements were resolved by consensus or adjudication by a third reviewer (LMAF). Overall study-level risk of bias was determined according to Cochrane algorithms.

### 2.8 Data synthesis

A narrative synthesis was performed for all included studies, structured to present and compare the characteristics of the populations, TMS intervention protocols (including frequency, intensity, duration, and cortical targets), comparator groups (where applicable), and reported outcomes. The synthesis focused on both primary outcomes—specifically, the presence and modulation of motor overflow—and secondary outcomes, such as motor function, spasticity, neurophysiological markers, and clinical relevance.

Studies were grouped by neurological condition (e.g., stroke, cerebral palsy, Parkinson’s disease, and dystonia) to facilitate condition-specific comparisons. Patterns were identified regarding TMS frequency (e.g., low vs. high), cortical targets (e.g., M1, SMA, PSC), and their differential effects on motor overflow and functional recovery. Attention was given to whether motor overflow was directly measured (e.g., via EMG, mirror movements, or kinematic proxies), and to the duration and clinical significance of observed effects.

Due to substantial heterogeneity in stimulation parameters, outcome definitions, and reporting formats, quantitative pooling through meta-analysis was not methodologically appropriate. Clinical and methodological heterogeneity was assessed qualitatively prior to synthesis.

Instead, findings were synthesized descriptively to highlight consistent trends, protocol-specific effects, and methodological gaps.

## 3 Results

**Figure 1:**
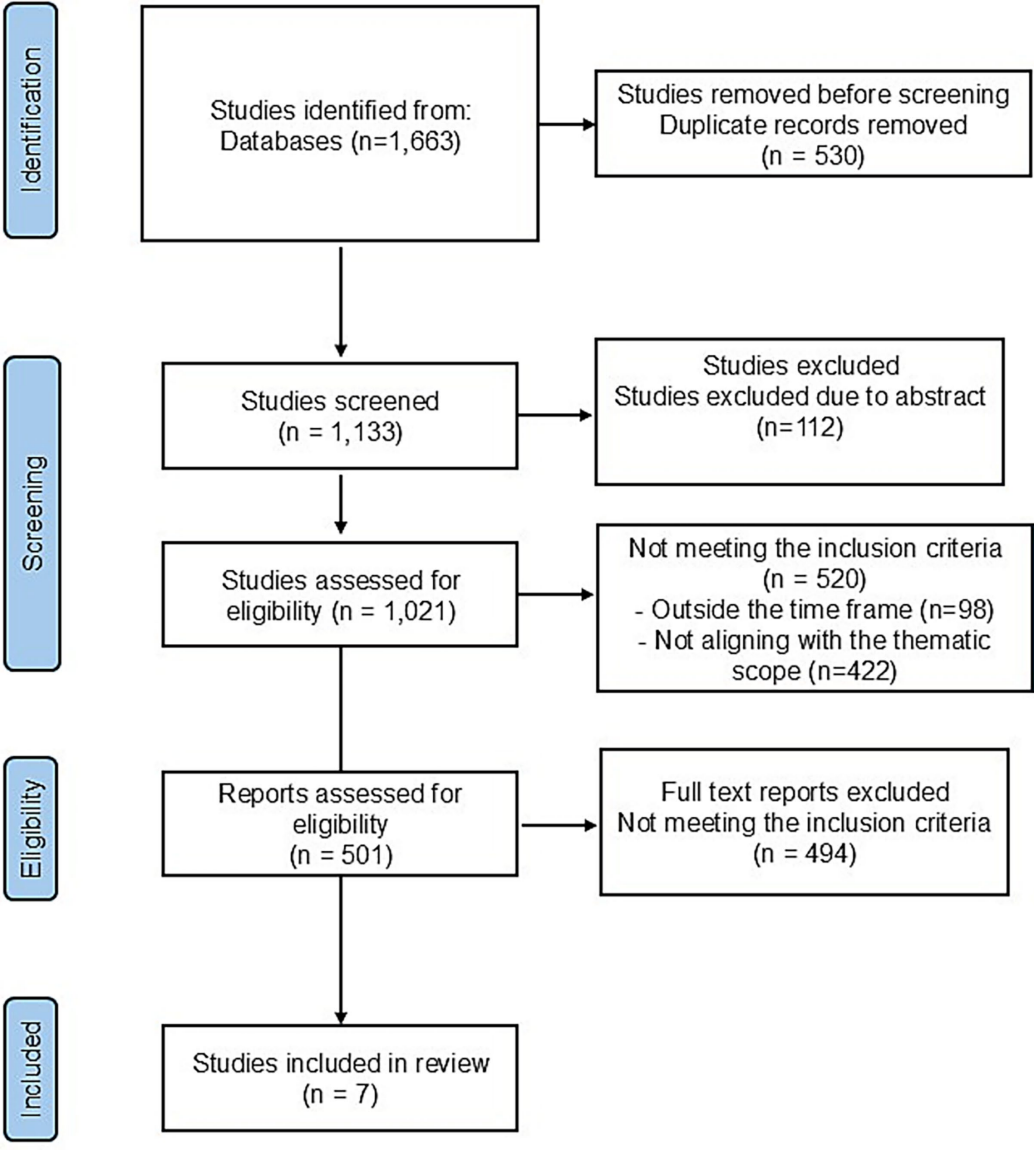
PRISMA flow diagram showing the selection process of studies included in the systematic review.

A systematic search in PubMed, Embase, Scopus, LILACS, and MEDLINE (via BVS) yielded 1,663 studies. After duplicate removal (*n* = 530), 1,133 records were screened by title/abstract and 501 full-text articles were assessed for eligibility. Ultimately, seven studies met the inclusion criteria. The PRISMA flow diagram summarizing this selection process is presented in Figure 1.

### 3.1 Study selection and characteristics

From an initial pool of 501 articles, 7 studies met the eligibility criteria and were included in this review.

The included studies represented diverse neurological populations, with three studies focusing on post-stroke motor impairment (Sung et al., 2013; Du et al., 2016; Li et al., 2021), two studies on cerebral palsy, one in children with spastic CP (Rajak et al., 2017) and one in adolescents with hemiparetic CP following perinatal stroke (Klingels et al., 2018), one study on Parkinson’s disease (Sharples et al., 2014), and one study on focal hand dystonia and writer’s cramp (Bukhari-Parlakturk et al., 2025).

All studies utilized repetitive transcranial magnetic stimulation (rTMS) protocols, with stimulation frequencies ranging from 1 Hz to 10 Hz. Specifically, low-frequency rTMS (1 Hz) was the most frequently applied (5 studies), particularly in stroke and CP populations (Sung et al., 2013; Du et al., 2016; Klingels et al., 2018; Li et al., 2021). High-frequency rTMS protocols (5–10 Hz) were used in Parkinson’s disease, CP, and dystonia (Sharples et al., 2014; Rajak et al., 2017; Bukhari-Parlakturk et al., 2025).

Moreover, two studies employed advanced stimulation paradigms: intermittent theta burst stimulation (iTBS) in post-stroke patients (Sung et al., 2013) and continuous theta burst stimulation (cTBS) in combination with low-frequency rTMS for stroke rehabilitation (Li et al., 2021).

Stimulation targets included key motor-related regions: the primary motor cortex (M1; Sung et al., 2013; Du et al., 2016; Rajak et al., 2017; Klingels et al., 2018; Li et al., 2021), the premotor cortex (PMC; Bukhari-Parlakturk et al., 2025), the supplementary motor area (SMA; Sharples et al., 2014), and the primary somatosensory cortex (PSC; Bukhari-Parlakturk et al., 2025).

Notably, the selection of associative and sensory regions (e.g., PSC and SMA) in dystonia and Parkinson’s disease reflects a mechanistic shift toward targeting sensorimotor integration networks rather than purely execution-related motor regions.

### 3.2 Primary outcomes: effects on motor overflow

#### 3.2.1 Dystonia

Motor overflow was most consistently evaluated in studies involving dystonia, where direct or proxy measures were employed. In patients with writer’s cramp, Bukhari-Parlakturk et al. (2025) demonstrated that 10 Hz rTMS targeted to the primary somatosensory cortex significantly reduced peak accelerations during handwriting tasks, indicating a measurable reduction in motor overflow and improved motor control at the session level.

This study represents the only included randomized controlled trial in which motor overflow was explicitly quantified as a primary behavioral endpoint and showed a statistically significant session-level effect.

#### 3.2.2 Parkinson’s disease

Sharples et al. (2014) evaluated mirror activation and interhemispheric inhibition (IHI) in Parkinson’s disease. While 5 Hz rTMS to the SMA reduced IHI, it did not change mirror movements.

This dissociation between neurophysiological modulation and behavioral expression suggests that cortical network changes do not necessarily translate into overt reductions in motor overflow, highlighting the complexity of overflow regulation in Parkinson’s disease.

#### 3.2.3 Stroke and cerebral palsy

Among individuals with stroke or cerebral palsy, five studies were reviewed (Sung et al., 2013; Du et al., 2016; Rajak et al., 2017; Klingels et al., 2018; Li et al., 2021). These studies used rTMS frequencies ranging from 1 Hz to 3 Hz and various cortical targets, including the primary motor cortex. Although these protocols consistently led to improvements in motor function, spasticity, and cortical excitability, none of the studies directly measured motor overflow or included it as a primary endpoint. Consequently, it remains unclear whether observed functional gains were accompanied by reductions in involuntary motor activation.

#### 3.2.4 Integrative synthesis of primary outcomes

Across neurological conditions, a clear pattern emerges: direct evidence for TMS-induced modulation of motor overflow is limited and condition-specific. Positive behavioral modulation was demonstrated primarily in dystonia using high-frequency stimulation targeting associative sensorimotor regions. In contrast, stroke and cerebral palsy trials focused on global motor recovery paradigms without explicitly quantifying overflow. Notably, only two of the seven included studies explicitly quantified motor overflow, whereas the remaining studies evaluated broader measures of motor recovery, cortical excitability, or functional performance. This finding highlights a substantial gap in the current literature regarding overflow-specific neuromodulation outcomes.

This discrepancy suggests that motor overflow modulation is not automatically achieved through general motor rehabilitation protocols and may require targeted cortical engagement combined with overflow-specific outcome measures.

### 3.3 Secondary outcomes: functional and neurophysiological changes

All seven studies included assessments of motor function or neurophysiology. The most common measures included the Fugl-Meyer Assessment (FMA), Modified Ashworth Scale (MAS), Barthel Index, and cortical excitability parameters (e.g., motor evoked potentials, short-interval intracortical inhibition).

Stroke studies consistently reported improved motor function with both low-frequency rTMS (1–3 Hz) and combined TMS protocols (Sung et al., 2013; Du et al., 2016; Li et al., 2021).

In hemiparetic cerebral palsy, Klingels et al. (2018) demonstrated that rTMS, combined with constraint-induced therapy, improved hand function and elicited favorable neurophysiological responses over a 6-month follow-up period. No adverse events or deterioration in motor performance were reported in any of the included trials.

However, the absence of standardized overflow-specific biomarkers (e.g., EMG asymmetry indices or quantitative mirror movement scales) limits interpretation of whether improvements reflected true restoration of motor selectivity or compensatory recruitment of alternative motor pathways.

### 3.4 Protocol-specific findings

#### 3.4.1 Frequency-specific effects

High-frequency transcranial magnetic stimulation (≥5 Hz) demonstrated the most consistent effectiveness in reducing motor overflow in individuals with dystonia, particularly when the stimulation was targeted at the primary somatosensory cortex (Bukhari-Parlakturk et al., 2025).

In contrast, low-frequency TMS (1 Hz) was predominantly used in studies involving stroke and cerebral palsy populations, where it produced broader functional improvements (Du et al., 2016; Rajak et al., 2017; Li et al., 2021).

#### 3.4.2 Combined protocols

Combined protocols emerged as promising strategies. For example, Li et al. (2021) demonstrated that coupling low-frequency rTMS with cerebellar continuous theta burst stimulation (cTBS) enhanced motor outcomes more than either intervention alone. Similarly, in children with cerebral palsy, Klingels et al. (2018) showed that the addition of constraint-induced movement therapy to rTMS significantly boosted upper limb functional gains.

These findings suggest that neuromodulation may exert additive effects when integrated with task-oriented rehabilitation, although the impact of such combined strategies on overflow-specific mechanisms remains unverified.

### 3.5 Measurement gaps and limitations

Direct measurement of motor overflow, such as surface electromyography (sEMG), mirror activation, or cortical silent period (CSP), was performed in only two of the seven included studies (Sharples et al., 2014; Bukhari-Parlakturk et al., 2025).

Clinical relevance of overflow reduction was clearly demonstrated only in the writer’s cramp study. In stroke and cerebral palsy populations, evidence remains indirect because outcomes focused on general motor recovery rather than direct quantification of motor overflow (Sung et al., 2013; Du et al., 2016; Rajak et al., 2017; Klingels et al., 2018; Li et al., 2021).

This heterogeneity, combined with the underreporting of overflow-specific outcomes in stroke and CP trials, substantially limits generalizability and prevents definitive conclusions regarding TMS as a targeted overflow-modulating intervention.

### 3.6 Risk of bias

The risk of bias for the seven randomized controlled trials (RCTs) included in this systematic review was assessed using the Cochrane Risk of Bias 2.0 (RoB 2) tool. This tool evaluates bias across five key domains: (1) bias arising from the randomization process, (2) bias due to deviations from intended interventions, (3) bias due to missing outcome data, (4) bias in measurement of the outcome, and (5) bias in selection of the reported result. The findings of the assessment were visualized using the ROBVIS (Risk Of Bias VISualization) tool.

**Figure 2:**
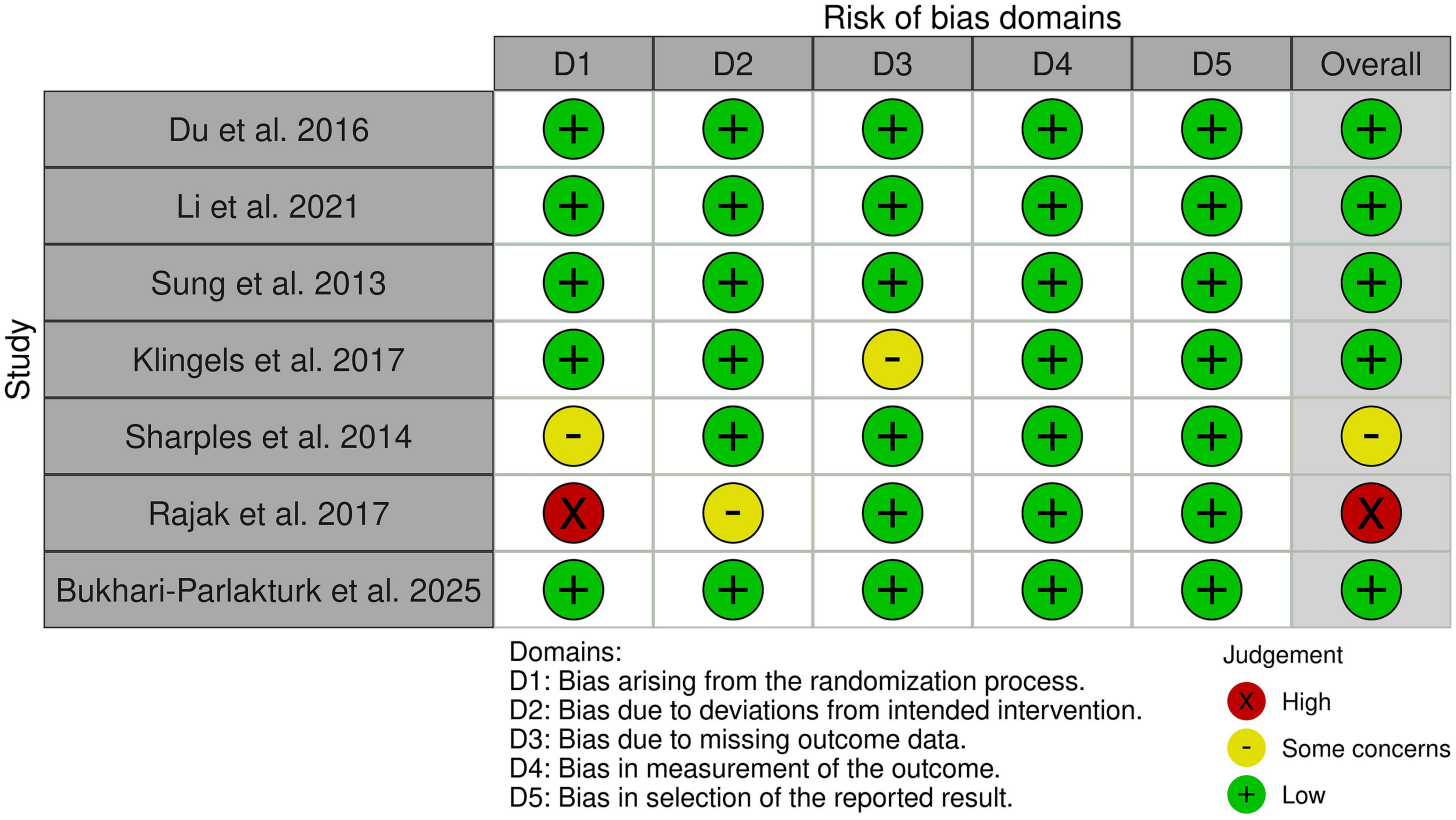
Risk of bias summary for the randomized controlled trials included in the systematic review.

As illustrated in Figure 2, five studies were judged to have “low risk” of bias across all domains (Sung et al., 2013; Du et al., 2016; Klingels et al., 2018; Li et al., 2021; Bukhari-Parlakturk et al., 2025). One trial was rated as having “some concerns” overall (Sharples et al., 2014), and one study was assessed as having “high risk of bias” due to multiple domains being flagged (Rajak et al., 2017).

Most studies demonstrated low risk in the domains of outcome measurement and selection of the reported result, reflecting adequate blinding of outcome assessors and sufficient transparency in reporting pre-specified endpoints. However, common issues were identified in the domain of the randomization process. For instance, Sharples et al. (2014) and Rajak et al. (2017) did not clearly describe allocation concealment procedures or random sequence generation, resulting in classifications of “some concerns” and “high risk” respectively. The domain concerning deviations from intended interventions raised some concerns in Rajak et al. (2017), as details on protocol fidelity or participant adherence were limited. Missing outcome data was adequately addressed in most trials; however, Klingels et al. (2018) did not clearly report attrition or dropout handling procedures, resulting in “some concerns” for that domain.

Overall, while methodological quality was generally acceptable, limitations in randomization transparency and protocol reporting underscore the need for more rigorously designed trials specifically targeting motor overflow as a primary outcome.

## 4 Discussion

This systematic review synthesized data from seven studies examining the effects of TMS on motor overflow in various neurological conditions. Overall, the evidence suggests that motor overflow can be modulated in dystonia under specific stimulation parameters, whereas in stroke and cerebral palsy populations, TMS has primarily been investigated for general motor recovery rather than overflow-specific outcomes. Importantly, one of the principal findings of this review is not that TMS has been conclusively demonstrated to reduce motor overflow across neurological disorders, but rather that direct investigation of motor overflow remains remarkably limited. Only two of the seven included randomized controlled trials explicitly quantified overflow-related outcomes, whereas the remaining studies focused primarily on motor recovery, cortical excitability, or functional performance. The results suggest that TMS can reduce motor overflow in focal dystonia, particularly writer’s cramp, when targeted at specific cortical regions such as the primary somatosensory cortex (PSC) using high-frequency protocols (10 Hz; Bukhari-Parlakturk et al., 2025). In contrast, while low-frequency TMS (1 Hz) applied to the contralesional hemisphere was commonly used in stroke and cerebral palsy populations, these studies did not directly assess motor overflow, instead focusing on general motor recovery and neurophysiological markers (Du et al., 2016; Klingels et al., 2018; Li et al., 2021). Only four of the seven included studies measured motor overflow through direct or proxy outcomes, which limits the ability to generalize findings across conditions. Importantly, this review highlights a central conceptual gap in the literature: motor overflow is frequently discussed as a clinical phenomenon, yet it is rarely operationalized as a primary therapeutic target in neuromodulation trials. Although these mechanisms are supported by the broader neurophysiological literature, they were not directly investigated in most of the studies included in this review and should therefore be considered explanatory hypotheses rather than confirmed mechanisms underlying the observed effects.

A notable finding is that in dystonia, motor overflow appears to be modifiable via specific TMS protocols, with high-frequency (10 Hz) stimulation over the PSC resulting in measurable reductions in abnormal muscle activation during fine motor tasks like handwriting. These effects were observed immediately after the session and in some cases persisted over multiple days (Bukhari-Parlakturk et al., 2025). However, these findings are based on small-sample crossover trials, and therefore should be interpreted as preliminary evidence rather than definitive proof of efficacy (Quattrone et al., 2023). From a mechanistic standpoint, this supports the hypothesis that dystonic overflow is strongly linked to maladaptive sensorimotor integration and deficient surround inhibition, processes in which the somatosensory cortex plays a critical modulatory role. Thus, PSC-targeted stimulation may exert its effects not merely by enhancing motor output, but by recalibrating aberrant sensory gating that contributes to involuntary co-activation.

Conversely, in stroke rehabilitation, TMS has primarily been employed to enhance motor recovery by rebalancing interhemispheric inhibition. The reviewed studies targeting stroke populations used 1 Hz inhibitory stimulation over the contralesional primary motor cortex or excitatory protocols (3–5 Hz) on the ipsilesional side, yielding improvements in Fugl-Meyer scores, muscle tone, and functional independence (Sung et al., 2013; Du et al., 2016; Li et al., 2021). The absence of direct overflow quantification in these trials represents a missed opportunity, given that impaired motor selectivity is a common feature after stroke and may contribute to suboptimal functional recovery (Kimberley et al., 2010). This disconnect suggests that improvements in global motor scores may not necessarily reflect restoration of selective motor control, and that overflow-related mechanisms may remain partially unaddressed even when functional gains are observed.

In cerebral palsy, particularly among children with hemiparesis, TMS has also shown promise in enhancing bimanual coordination and upper limb function when combined with constraint-induced movement therapy (Rajak et al., 2017; Klingels et al., 2018). However, standardized overflow-specific outcomes were not included. Given that mirror movements are highly prevalent and clinically relevant in unilateral CP, the omission of overflow-specific outcomes limits the interpretability of the reported functional improvements. Future trials incorporating surface EMG-based mirror activation metrics or interhemispheric inhibition indices could provide clearer insight into whether functional gains are accompanied by improvements in motor selectivity.

From a clinical perspective, motor overflow presents challenges in functional rehabilitation, as it reduces the selectivity and efficiency of movement. In physiotherapy, techniques like Proprioceptive Neuromuscular Facilitation (PNF) or bilateral task training aim to enhance selective motor control and interhemispheric balance (Nachev et al., 2008; Shenhav et al., 2013). Recent randomized controlled trials in healthy adults have demonstrated that contralateral activation of proximal muscles, such as the hip flexors, can attenuate the decline in handgrip strength during repetitive tasks, suggesting a neural facilitation effect with potential application in neuromuscular rehabilitation (Rosa et al., 2025) Integrating TMS with task-oriented rehabilitation may therefore represent a strategy to simultaneously modulate cortical excitability and reinforce motor selectivity through use-dependent plasticity. This integrative model—combining neuromodulation with physiotherapeutic motor retraining—constitutes a novel conceptual implication emerging from this review.

Importantly, only a minority of studies included neurophysiological outcome measures, such as CSP, IHI, or MEP latency/amplitude, to support the behavioral findings. These tools could offer objective biomarkers of overflow and help differentiate between functional improvement and compensatory strategies. For instance, Sharples et al. (2014) demonstrated that 5 Hz stimulation of the SMA reduced interhemispheric inhibition but did not improve mirror movements in Parkinson’s disease, underscoring that modulation of cortical networks may not always result in observable motor benefits. This dissociation between physiological modulation and behavioral expression underscores a key theoretical insight: normalization of cortical excitability does not automatically translate into restoration of selective motor control. Future trials must therefore integrate multimodal outcomes to capture both neural and behavioral dimensions of overflow.

The heterogeneity in study design, stimulation parameters (frequency, target, intensity), and outcome assessments is a critical limitation. This diversity reflects the exploratory nature of TMS research in motor overflow but also impairs data comparability. Our synthesis suggests that protocol specificity—particularly frequency and cortical target—may be more relevant than condition alone in determining overflow modulation. Standardized protocols and consensus on core outcome sets, similar to those proposed for post-stroke motor trials, are needed to establish reliable clinical recommendations (Kwakkel et al., 2017).

Additionally, small sample sizes and limited follow-up periods were common across studies, particularly in trials for dystonia. Although positive effects on overflow were noted, long-term maintenance, generalization to daily function, and transfer across tasks remain poorly understood. Without longitudinal data, it is impossible to determine whether observed reductions in overflow represent transient modulation or durable reorganization of motor networks. Until larger, adequately powered randomized controlled trials are conducted, TMS should be considered a promising adjunctive strategy rather than a stand-alone intervention for motor overflow.

Several limitations must be considered when interpreting the findings of this review. First, the included studies were characterized by small sample sizes, heterogeneous stimulation parameters (frequency, intensity, cortical targets), and short follow-up periods, which limit statistical power and long-term generalizability. Additionally, there was considerable variability in outcome measures, and most studies conducted in stroke and cerebral palsy populations did not directly quantify motor overflow, instead focusing on general motor recovery or neurophysiological markers. This limits the ability to draw firm conclusions regarding overflow-specific effects of TMS in these conditions. Furthermore, methodological inconsistencies in randomization reporting, blinding procedures, and protocol fidelity may have introduced bias in some trials. At the review level, the restriction to published peer-reviewed RCTs may have introduced publication bias, and language limitations could have excluded relevant studies. The substantial heterogeneity in study design and measurement tools precluded quantitative meta-analysis, necessitating a narrative synthesis approach. Collectively, these limitations underscore the need for larger, well-designed trials with standardized overflow-specific outcome measures to strengthen the evidence base. Another limitation is that most included studies were not originally designed to evaluate motor overflow as a primary outcome. Consequently, several conclusions regarding overflow-related mechanisms are necessarily indirect and should be interpreted cautiously. Additionally, although the search strategy included the principal descriptors related to motor overflow, studies using alternative terminology (e.g., mirror activity, associated movements, synkinesis, motor irradiation, or involuntary co-activation) may not have been identified.

This systematic review provides preliminary evidence that TMS may modulate motor overflow, particularly in focal dystonia when high-frequency protocols are applied to the primary somatosensory cortex. However, this conclusion is based on a limited number of studies directly assessing motor overflow and should therefore be interpreted cautiously. However, this conclusion is primarily based on small-sample crossover trials and should be interpreted cautiously. In populations with stroke and cerebral palsy, TMS has predominantly been investigated as a strategy for general motor recovery, and because motor overflow was not directly assessed in most studies, no definitive conclusions can be drawn regarding its modulation in these conditions. Furthermore, a formal assessment of certainty of evidence using the GRADE framework was not performed, as it was not specified in the original review protocol. Consequently, the overall certainty and strength of the available evidence should be interpreted with caution.

The principal conceptual contribution of this review lies in identifying motor overflow as an under-defined yet clinically relevant neuromodulation target, distinct from general motor recovery. At present, the available evidence is insufficient to support routine clinical application of TMS specifically for overflow reduction. Future studies should explicitly define motor overflow as a primary endpoint, incorporate standardized and objective measurement tools, and employ adequately powered randomized controlled designs to clarify whether targeted neuromodulation can meaningfully and sustainably improve motor selectivity.
